# Evaluation of the RISE II integrated social and behavior change approach in Niger: A contribution analysis

**DOI:** 10.1371/journal.pone.0308185

**Published:** 2024-07-31

**Authors:** Leanne Dougherty, Chaibou Dadi, Martha Silva

**Affiliations:** 1 Population Council, Washington, DC, United States of America; 2 Conception Etudes Suivi Evaluation Appuis Formation, Niamey, Niger; 3 Tulane University, New Orleans, LA, United States of America; Uniformed Services University: Uniformed Services University of the Health Sciences, UNITED STATES OF AMERICA

## Abstract

**Objective:**

Niger faces a myriad of health challenges and development efforts are complicated by persistent poverty, high population growth rates, and climate change. Integrated social and behavior change (SBC) addresses health outcomes through collective action and approaches at the limited points of entry individuals have with the health system.

**Methods:**

We conducted a mixed-methods study to evaluate the effectiveness of an integrated SBC program in the Maradi, and Zinder regions of Niger. We applied contribution analysis, a theory-based plausibility analysis, to assess contributions of the intervention.

**Results:**

We found the program contributed to improved behavioral determinants. Male engagement and income generating activities provided further support for women to practice health behaviors. However, increases in male partner out-migration was negatively associated with health outcomes. While the program did not generate statistically significant improvements in health outcomes in the intervention area, exposure to health messages and participation in women’s groups were positively associated with health outcomes suggesting sustained implementation of the integrated SBC approach at scale may achieve improved health outcomes.

**Conclusion:**

Programs should continue to invest in health promotion efforts that include gender sensitive interventions. Further research is needed to understand how women’s agency and autonomy evolves as household composition changes through male out-migration.

## Introduction

The Sahel region experiences persistent poverty, recurrent climate shocks, high population growth rates and food insecurity. In the region, 2.7 million people are internally displaced due to violence and persecution, and 29 million require humanitarian assistance, including 1.6 million children who are severely malnourished [1]. In Niger, located within the Sahel, a high proportion of children are stunted (44%) [2] and under-five mortality rates are high (84 per 1,000) [3]. The maternal mortality ratio was 509 per 100,000 live births in 2017 [4] and according to the most recent survey, the total fertility rate was 6.2 in Niger in 2021[5]. Pro-natalist cultural norms coupled with a lack of decision-making power by women for health and fertility matters further challenge efforts to improve health outcomes contributing to low levels of modern contraceptive use (12%) [2].

In Niger, there is an increased interest in building on existing humanitarian and development assistance to provide cross-sectoral funding that cohesively addresses more than one health or development issue within the same program. Integrated social and behavior change (SBC) addresses multiple health outcomes through collective action and approaches (e.g., community engagement, interpersonal communication, etc.) at the limited points of entry for individuals to interact with the health system (in the community and/or at a facility) [6]. Integrated approaches are particularly useful in rural resource-constrained settings with limited accessibility to the health system and with populations that are mobile (e.g., pastoralists). In addition, many healthy behaviors have benefits across multiple health outcomes allowing programs to reach people with multiple health messages through a single intervention. For example, improved hygiene and access to potable water can lead to improved nutritional outcomes and reduce the risk of diarrheal diseases. Among the studies that have assessed integrated SBC programs in the Sahel, several incorporated a gender sensitive approach and found female empowerment and male engagement activities contributed to the effectiveness of the program [7–9]. While there is some evidence that integrated SBC programming in the Sahel is effective at changing behaviors, very few large scale studies support this finding and available evidence is not rigorous [10–12].

### Intervention description

The United States Agency for International Development’s (USAID) supported Resilience in the Sahel Enhanced (RISE) II project targeted chronically vulnerable populations through integrated SBC programming to improve priority behaviors and health outcomes in family planning (FP); maternal, newborn, and child health (MNCH); nutrition; and water, sanitation, and hygiene (WASH). The program was implemented through the Resilience Food Security Activity (RFSA) partners in select zones in Maradi and Zinder regions of Niger from 2019–2023. The RFSA partners in Niger included Hamzari (led by Care), Girma (led by Catholic Relief Services), and Wadata (led by Save the Children). Prior to implementation in 2019, RFSAs received support from USAID for a yearlong refine and implementation period to inform activity design through stakeholder engagement, formative research, and theory of change development. RFSAs used a variety of SBC approaches to address factors influencing health outcomes at the individual, interpersonal and community levels. At the individual level, RFSAs used Care Groups and home visits. Volunteer leaders identified pregnant women and children up to six months of age in the village and trained facilitators conducted monthly community based peer group meetings and conducted home visits to share messages that encouraged these women to visit health centers for prenatal consultations before childbirth, to opt for skilled birth attendants, to adopt optimal lactating practices following birth and to improve hygiene practices through the use of locally made, low cost handwashing stations that motivate handwashing at critical moments [13,14]. The Care groups relied on cascade training from paid promoters to volunteer leaders to neighborhood mothers to enable broad geographic coverage. At the interpersonal level, similar community-based peer groups were organized for men through Husband’s schools. Through the Husbands Schools model, men educated their peers on issues relating to sexual and reproductive health, and child nutrition. Through both Care groups and Husband’s schools, RFSAs encouraged intra-couple dialogue as a lever of behavior change to promote joint decision making for health behaviors particularly related to contraceptive use and maternal health care. At the community level, RFSAs also worked with community leaders and through radio programs to reinforce health messages. Radio programs developed by RFSAs served as a platform where members in the community debated social issues and addressed social norms such as early pregnancy among young married adolescents. In addition to the primary SBC strategies, RFSA’s supported community group activities to enable income generation.

### Theory of change

**Fig 1:**
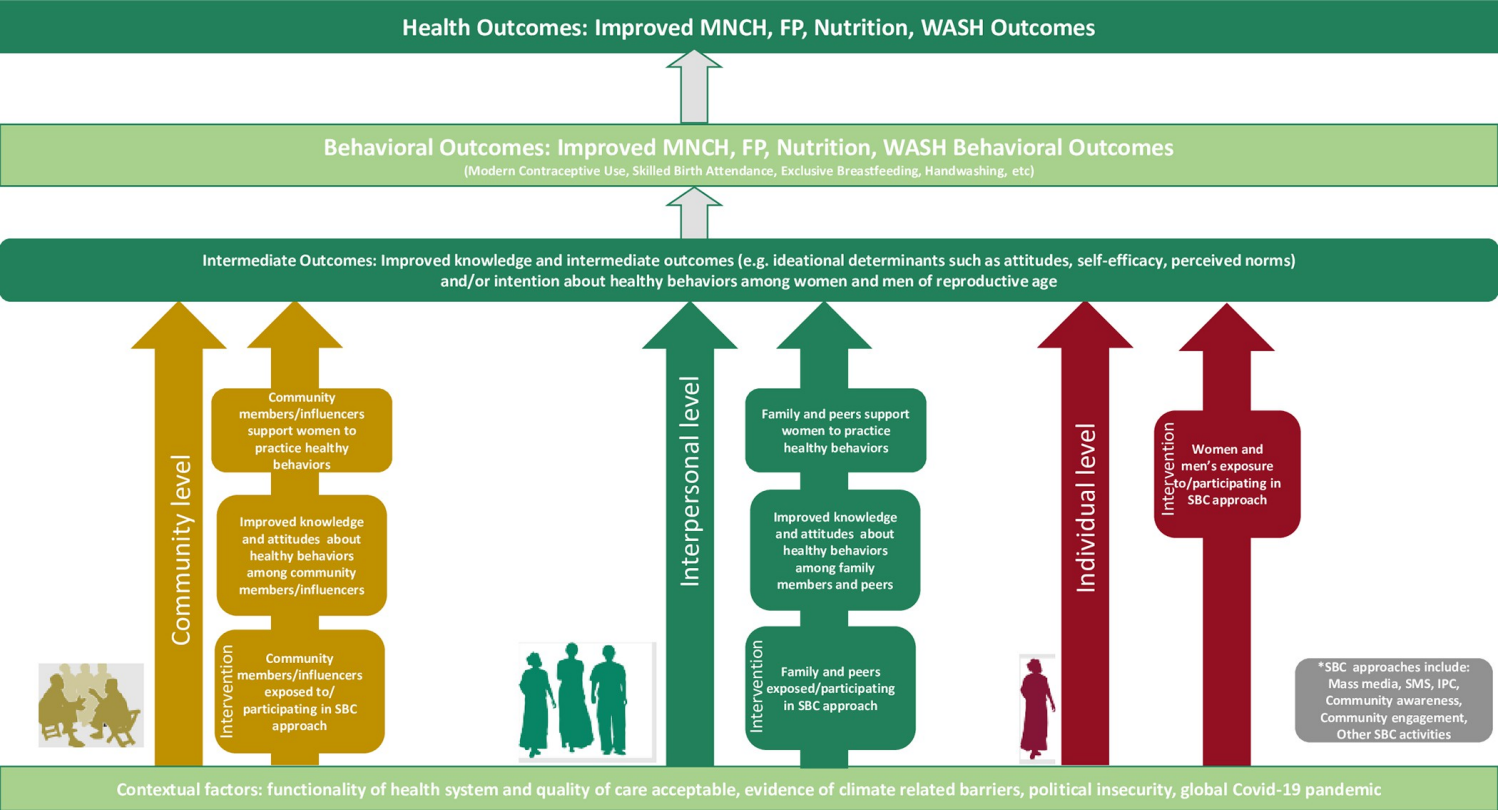
RISE II integrated social and behavior change theory of change.

Fig 1 presents a theory of change that describes how the RISE II SBC activities were expected to influence health behaviors. The theory of change is guided by the socio-ecological model [15], which recognizes the influence of factors operating at five levels: 1) individual, 2) interpersonal, 3) community, 4) health service delivery, and 5) policy. The RFSAs addressed factors operating at the individual, interpersonal, and community levels. The SBC approaches targeted not only individual women and men but also family and peers, community members, and influencers because social relationships within a community have a strong influence on health behaviors [16]. The SBC activities aimed to improve knowledge, intermediate outcomes (e.g., ideational determinants such as attitudes, and norms) and intention to adopt healthy behaviors by taking advantage of the social structural factors that influence behavioral choices, including relationships and equitable availability of social and material resources. By shifting health -related behavioral determinants of key members of the community, RISE II partners anticipated improved health outcomes.

In this paper, we assess the effectiveness of integrated SBC programming on FP use, maternal health, nutrition, and WASH behaviors. Guided by the theory of change, we consider the mechanisms through which changes occurred by considering behavioral determinants and their association with health behaviors among women of reproductive age as well as contextual factors that influence programmatic impact.

## Material and methods

### Study area and design

We conducted a mixed-methods triangulation study drawing from a pre-post quasi-experimental survey and qualitative in-depth interviews with program participants to evaluate the effectiveness of the RISE II integrated SBC program in the Maradi and Zinder regions of Niger. Maradi includes the city of Maradi, the second largest in Niger, but overall is quite rural. Zinder is very rural and extends north into the Sahel. Households in these districts rely on rainfed agriculture and pastoralism for their livelihoods. The baseline quantitative survey started in April and concluded in early May 2021 with a final sample of N = 2,708. The survey was conducted one year after program implementation was underway due to Covid-19 related delays that were imposed to protect the health and safety of human subjects. The endline survey was conducted in February 2023 and concluded in March 2023 with a final endline sample of N = 2,727. Qualitative interviews were conducted in June 2022.

### Study procedures

For both the quantitative and qualitative data collection efforts, male interviewers interviewed men, and female interviewers interviewed women because Islamic cultural practices require limited interaction between sexes if individuals are not related. Trained interviewers described the objectives of the research, obtained written informed consent from all participants (or verbal consent if the participant was unable to write), and then administered the study instruments in Hausa to the study participants. Interviewers were fluent in both French and Hausa.

### Quantitative

**Fig 2:**
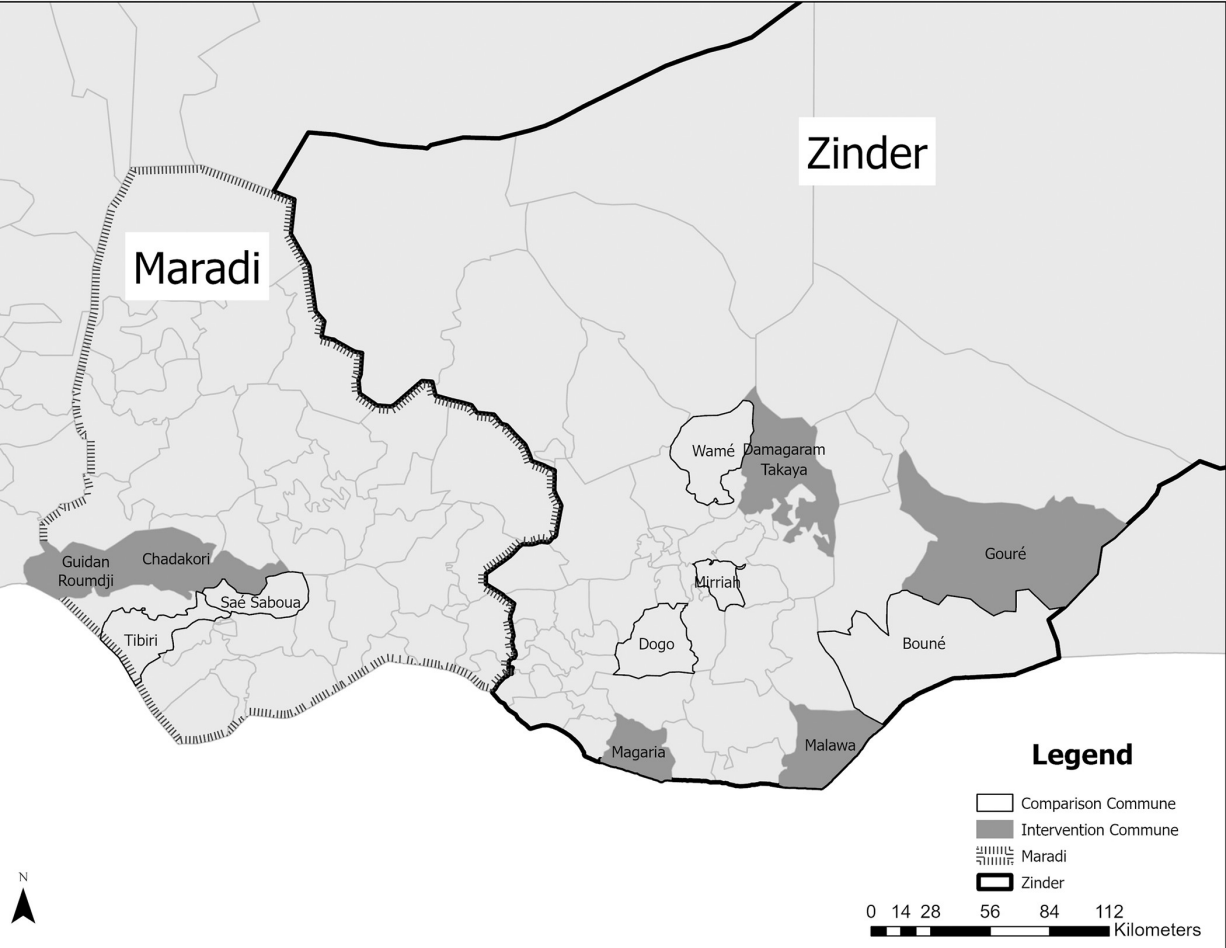
Map of Niger and RISE II study areas.

We applied a three-stage stratified sampling procedure. In the first stage, we randomly selected six intervention communes from the 18 intervention communes and six comparison communes. In both the intervention and comparison areas we selected four communes in Zinder and two communes in Maradi. The comparison communes were selected based on similar socio-demographic characteristics, population density and healthcare accessibility. A map of the study area is shown in Fig 2.

In the second stage, we listed all enumeration areas (EA) identified in the 2012 General Census in each of the communes and used probability proportion to size to select 40 EAs for each study group. In the third stage, for the intervention group, the study team used a household listing completed by the implementing partners to identify all households with eligible women (married women between 15 and 49 years of age). For the comparison group, the study team enumerated all households in each of the selected EAs to identify households with eligible women. We then randomly selected 34 households per EA accounting for a 10% non-response rate to achieve the final sample size. This sample size was based on a minimum detectable difference of 6–11 percentage points in the priority indicators between study groups, with 80% power to detect a difference and alpha of 0.05.

### Study outcomes and covariates

We selected four indicators to assess the effectiveness of the integrated SBC approach on reproductive, maternal, nutrition and WASH behaviors [17]. To assess use of family planning, we measured the modern contraceptive prevalence rate (MCPR). To assess maternal health, we measured the percentage of women in the two preceding years who delivered in a facility for their last birth. For nutrition, we measured the percentage of children between 0 and 5 months who were exclusively breastfed in the 24 hours preceding the survey and finally to assess hygiene behaviors, we measured the percentage of households with a handwashing station as a proxy for handwashing behavior. Drawing from the program theory of change, we measured knowledge, attitudes, perceived norms, and self-efficacy for each of these health outcomes. We also measured demographic and contextual related factors including a wealth index using Equity tool methods for Niger [18], whether the woman listened to the radio or watched television, age, education, marital status (monogamous or polygamous in the ever married sample), experience with family migration (e.g., does your husband/partner currently live with you or does he live somewhere else?) and drought (e.g., in the past 12 months has your village experienced a drought–that is a lack of rainfall for a period of time that resulted in less available water than is required to meet the needs of your community?). Finally, we assessed exposure to SBC programming by assessing whether the woman attended a woman’s group organized by the RFSA partner and whether they were exposed to a health message (e.g., family planning) in the three months prior to the survey. Notably, the southern region of Niger is saturated with development projects including in the comparison study area. We administered questions on exposure to SBC programs to both study groups to account for potential exposure to health messages outside the intervention zone.

### Qualitative

The qualitative component of the study used in-depth interviews with program participants to assess how the program was implemented and to what extent conditions during implementation influenced the results. Seven men and seven women were selected from each intervention commune for a total of 21 female and 21 male program participants (see Fig 2). Study participants were selected randomly from a roster of RISE II intervention participants provided by the RISE II implementing partners. Trained interviewers conducted in-depth interviews until saturation was achieved. Interviews were conducted in person in Hausa and recorded using a digital audio recorder with permission from study participants. Recordings were translated and transcribed into French for analysis.

### Analysis

First, we conducted bivariate chi-squared analyses for all measures of interest by time (baseline vs. endline), and study group (intervention vs comparison). Next, to measure the effect of the program on intermediate behavioral determinants, we estimated simple logistic regression models while controlling for socio-demographic and contextual factors as well as exposure to the intervention, time, study group and an interaction term (time*study group). Finally, for the health outcomes, we estimated simple logistic regression models that included the intermediate behavioral outcomes, socio-demographic and contextual factors, exposure to intervention, time, study group and the interaction variable. Analyses were conducted using STATA 16 SE [19]. The measures of exposure to the intervention included a measure to assess whether the study participant had participated in a RFSA sponsored woman’s group and whether they had been exposed to health specific messages. We assessed the variance inflation factor for each model and confirmed variable independence. We accounted for the design effect by assigning the EAs as the primary sampling unit for the analysis and stratifying by commune.

To analyze the qualitative data, we developed codes based on the theory of change including intervention components, intermediate outcomes (e.g., knowledge, attitudes) and contextual factors (e.g., climate, disease outbreaks, etc.). Additional codes were developed by applying open coding on a subset of transcripts to co-construct new themes. We applied codes using NVivo 12 [20]. Prior to applying the codes across all transcripts, a subset of transcripts was coded by the team (the authors of this manuscript) to ensure agreement in coding. Inconsistencies were resolved following discussion with the study team. We used thematic analysis to compare findings across study participant type (e.g., male vs female and by RFSA partner) to highlight divergent and convergent themes. All coding and analysis were conducted in French with data excerpts translated to English for publication purposes.

Contribution analysis (CA) is a theory-based plausibility analysis that can be helpful in assessing contributions of complex interventions [21]. The goal of the CA is to reduce uncertainty about the contribution an intervention is making towards results by describing why results did or did not occur and the roles played by the intervention as outlined in the theory of change and other influencing and external factors. CA is based on a postulated theory of change for the intervention being examined and tests this theory of change using multiple sources of data to measure intervention exposure, intermediate outcomes, perception of program performance and external factors. To set out the cause-and-effect issue to be addressed and develop the theory of change we reviewed the RFSA programmatic documents and held interviews to discuss their planned activities. We gathered evidence to assess mechanisms in the theory of change and applied Lemire’s framework, the Relevant Explanation Finder (REF), to test the contribution story and identify any challenges to its validity [22]. The REF framework allowed us to operationalize the CA by defining the theory of change mechanism and level and the type of explanation (e.g. Primary explanation–a mechanism identified and purported to be the target intervention mechanism that accounts for and explains the observed outcomes; Commingled rival–other mechanisms, along with the target mechanism, that both contribute to and explain the observed outcomes; Implementation rival–influencing factors in the implementation process, not substantive intervention mechanisms, that modify the outcomes). We then developed a contribution story identifying elements of the intervention that contributed to the health outcomes.

## Results

Table 1 provides a description of study participants at baseline and endline by study arm. The groups were similar across socio-demographic characteristics at baseline except for age where the comparison group was slightly younger than the intervention group. At endline, we found statistically significant differences related to women reporting their husband living away from the home and more women reporting being in monogamous relationships in the comparison areas. We also found that study participants were more likely to be exposed to messages related to FP, maternal health, nutrition, and WASH messages in intervention areas compared to comparison areas, which is to be expected.

**Table 1:** Description of study participants (quantitative surveys).

	Baseline			Endline		
	Intervention N = 1,353 (%)	Comparison N = 1,355 (%)	p-value	Intervention N = 1,360 (%)	Comparison N = 1,367 (%)	p-value
**Wealth status**			0.66			0.17
Poorest	33.8	33.7		31.9	39.7	
Middle	34.7	31.3		31.2	30.5	
Wealthiest	31.6	35.1		36.9	29.8	
**Last 12 months experienced a drought**			0.11			0.47
Yes	50.9	46.4		8.9	7.7	
**Husband lives away**			0.90			0.00
Yes	6.1	5.9		8.5	14.0	
**Age group in years**			0.03			0.41
15–24	13.8	19.3		25.4	27.9	
25–34	51.8	48.1		50.1	49.7	
35–49	34.4	32.6		24.5	22.4	
**Educational attainment**			0.24			0.09
No education	86.8	84.1		80.0	84.6	
Some formal schooling	13.2	15.9		20.0	15.4	
**Marital status**			0.51			0.03
Married monogamous	61.6	63.3		56.3	62.7	
Married polygamous	38.4	36.7		43.8	37.3	
**Attends woman’s group**			0.11			0.18
Attends woman’s group	32.0	26.5		19.6	16.0	
**Watch television**			0.97			0.38
At least once a week	7.2	7.1		4.8	3.2	
Less than once a week	92.8	92.9		95.2	96.8	
**Listen to radio**			0.16			0.00
At least once a week	31.0	35.0		25.5	16.5	
Less than once a week	69.0	65.0		74.5	83.5	
**In the past 3 months, have you heard or seen any messages…**						
about family planning	60.8	57.9	0.37	48.5	38.4	0.00
related to seeking care at a facility during pregnancy or childbirth?	63.6	64.7	0.63	51.2	41.9	0.00
related to breastfeeding and nutrition?	62.5	60.8	0.49	48.7	32.1	0.00
related to handwashing?	65.3	62.3	0.25	46.0	25.8	0.00
**Behavioral Outcomes**						
MCPR	26.5	23.0	0.15	35.0	28.7	0.07
Handwashing station	68.1	67.5	0.65	54.9	57.4	0.25
	Intervention N = 1,278 (%)	Comparison N = 1,258 (%)	p-value	Intervention N = 1,360 (%)	Comparison N = 1,367 (%)	p-value
Facility Delivery	56.0	56.1	0.97	49.9	45.4	0.39
	Intervention N = 274 (%)	Comparison N = 265 (%)	p-value	Intervention N = 318 (%)	Comparison N = 334 (%)	p-value
Exclusive Breastfeeding	24.2	25.0	0.83	46.8	41.4	0.17

The findings are organized as follows. First, we summarize evidence by the socioecological levels (e.g., individual, interpersonal and community) outlined in the theory of change and then describe changes in the intermediate outcomes related to FP, maternal health, nutrition, and WASH, followed by the effects of the program on health outcomes. We use the qualitative data to describe contextual factors that may be influencing program outcomes at these levels. We then use the REF to bring together the multiple sources of data to consider the theory of change mechanisms, alternative explanations and influencing factors, the degree of influence and the implications. Based on this analysis, we conclude with the contribution story.

### Individual level

An important element of the RFSA approach was the use of Care groups, which relied on cascade training from paid promoters to volunteer leaders to neighborhood mothers to enable broad geographic coverage. Neighborhood mothers shared information through community meetings, door-to-door sensitizations and through community events such as funerals, baptisms, and wedding ceremonies.

*"Before, a pregnant woman did not go to the prenatal consultation, but with the arrival of the project things have changed. The project comes to target us to bring us to the training in town and when we come back we sensitize the others too. Now, everyone has understood that as soon as a woman becomes pregnant and has reached three months of pregnancy, she must start going to the prenatal consultation… . " Woman Girma*

Several respondents who participated in the training reflected on challenges experienced. Some women were resistant to engage with the volunteers on health messages because they felt they should receive something in exchange, but this was resolved through dialogue.

*"When we were brought to the training, we went door to door to sensitize the women. There are 14 women under my care, I go back to sensitize them and it happened that the women said that we don’t give them anything, we only come to discuss with us. that’s when we almost had a problem, that too now that they have understood, they have seen that it’s their health that we’re taking care of, it’s not something*," *Woman Girma*

The quantitative data measured levels of exposure among women to FP, maternal health, nutrition, and WASH messages at baseline and endline by study arm (Table 1). We found comparable levels of exposure to messages by health area at baseline between intervention and comparison areas with approximately 60% of study participants stating that they had heard or seen messages in the past three months. At endline, approximately 50% of women stated they had seen or heard a health message in the last three months. In the comparison area, exposure to messages was as low as 25% among women had heard or seen a message related to handwashing and approximately 42% who had heard or seen a message related to pregnancy and childbirth. Across all health messages, women were significantly more likely to have heard or seen a health message in the intervention group. Similar trends were observed among women attending Care group activities.

### Interpersonal level

Findings from the qualitative data explored exposure to the SBC approach among family and peers and how this contributed to improved knowledge and attitudes about health behaviors among program participants. Respondents reflected on the RFSA sponsored community activities and how the program engaged husbands and wives. This approach helped to facilitate communication between couples and even the broader family to identify solutions to household problems.

*"The sensitization activity on the roles of husbands is the most useful for me…because the husband and wife discuss together the problems of the household and find solutions. You discuss together, even with the children, and each one gives his point of view*" *Male, Wadata*

Evidence from the qualitative data also considered the extent to which family members and peers supported women to practice healthy behaviors. Several respondents reflected on how the project encouraged husbands to support women with household work.

*"We were told that if you are overwhelmed with housework, your husband just has to help you… Even if it’s the dishes, or the children’s toilet, if the wife is overwhelmed, we say the husband has to help her. It’s a way of supporting each other…And you don’t want to have children to the point where some are sitting, one is on the back, the other is breastfeeding. They say that this is poverty*” *Woman, Wadata*

Several women reflected on the value of income generating activities in enabling them to access health services without relying on their partner for financial support.

*"And then with my business activity, I will rush to go to a health center whether it is me or my children, I will not rely on my partner….." Woman Hamzari*

### Community level

Findings from the qualitative data explored community members’ exposure to the SBC approach and how this contributed to improved knowledge and attitudes about health behaviors in the community and support of women to adopt healthy behaviors.

Radio programs served as a channel to share health messages. However, as noted in Table 1, fewer than a third of women interviewed listened to the radio at least once a week at baseline. While access to radios was limited, several program participants reflected on hearing health messages transmitted through radio programs.

Several program participants noted that health information was available through the radio, television, project, and the health center. The availability of health information through mass media and the health center suggests communities outside the intervention areas were also likely reached through broader government health strategies.

*“information? [Noise from recording], to [ok] we hear the information on the radio, we hear it on TV, we hear it through this project. They came to gather us, they make us, we hear it at the health center too……*" *Woman Zinder*

*Knowledge and intermediate outcomes related to FP*, *maternal health*, *nutrition and WASH*

The quantitative data measured knowledge and intermediate outcomes among women of reproductive age (Table 2).

**Table 2:** Key indicators according to the theory of change as reported by married women of reproductive age at baseline and endline, Niger 2021–2023.

	Baseline			Endline			Interaction (Time*Study group)	
	Interven-tion N = 1,353 (%)	Compari-son N = 1,355 (%)	p-value	Interven-tion N = 1,360 (%)	Compari-son N = 1,367 (%)	p-value	aOR* [CI]	p-value
**Family Planning**								
Know: Has ever heard of at least 3 different FP methods	77.6	79.4	0.47	86.4	84.4	0.49	1.17 [0.69–1.97]	0.56
Att: Agree it is acceptable for a couple to limit the number of children they have	48.3	49.2	0.73	38.5	36.0	0.16	1.16 [0.89–1.52]	0.28
Norm: Agree that religious leaders support a woman’s use of FP	50.9	51.6	0.84	62.1	60.5	0.52	1.01 [0.68–1.49]	0.96
SE: Agree they are comfortable discussing FP with their partner	62.0	66.4	0.15	62.6	56.8	0.02	1.39 [0.90–2.14]	0.13
**Facility Delivery**								
Know: Knows that a woman should give birth at a health facility	91.9	94.3	0.11	93.5	90.0	0.07	1.97 [1.00–3.90]	0.50
Att: Agree it is important for a woman to discuss her pregnancy with her husband so they make decisions together	89.7	91.5	0.23	96.3	95.1	0.36	1.47 [0.72–2.99]	0.29
Norm: Agree that most women in the community give birth at a health facility	62.3	72.0	0.05	56.1	52.3	0.49	1.49 [0.78–2.85]	0.23
SE: Agree it is not difficult to get to a health facility to give birth	79.2	80.0	0.82	61.5	57.7	0.43	0.88 [0.47–1.64]	0.68
**Exclusive Breastfeeding**								
Know: Knows it is healthy to give only breastmilk for the first 6 months	68.6	66.7	0.44	75.3	61.7	0.00	1.42 [0.96–2.11]	0.01
Att: Agree if baby is exclusively breastfed for 6 months they are less likely to be sick	88.1	89.2	0.54	92.1	86.5	0.01	1.87 [1.04–3.34]	0.04
Norm: Agree people in the community think it is healthy for a woman to give the baby only breastmilk for the first six months	49.7	45.6	0.18	59.6	45.7	0.00	1.77 [1.19–2.63]	0.01
SE: Agree giving only breastmilk to the baby for the first 6 months is not difficult at all	64.5	61.2	0.21	62.7	47.8	0.00	1.16 [0.70–1.92]	0.56
**Handwashing Station**								
Know: Know 3 critical times to wash hands	44.1	35.9	0.00	27.0	22.0	0.02	0.54 [0.40–0.72]	0.00
Att: Disagree washing hands with soap before eating will ruin the taste of the food	83.2	89.7	0.00	96.8	95.5	0.06	2.88 [1.56–5.3]	0.00
Norm: Agree people in this community wash their hands after defecating	81.2	80.5	0.76	72.7	70.1	0.24	5.01 [3.25–7.73]	0.00
SE: Agree washing hands with soap after defecating is not difficult at all	70.1	83.6	0.00	93.8	87.2	0.00	0.81 [0.54–1.23]	0.32

Know (Knowledge; Att (Attitude); Norm (Perceived Norm); SE (Self efficacy).

*Controlling for wealth, exposure to drought, husband lives away, age, educational attainment, marital status, listens to radio, watches TV, exposure to messages, participates in women’s group.

Significance: *p = < 0.05; **p = < 0.01; ***p < 0.001.

At baseline, there were no statistically significant differences across all intermediate outcomes for family planning. Improvements in knowledge, perceived norms and self-efficacy were observed at endline but were not statistically significant. There was a statistically significant difference in the association between women who agreed they felt comfortable discussing family planning with their partner at endline by study group. However, when controlling for the interaction term (e.g., time and study group), there was not a statistically significant association.

For the facility delivery behavioral determinants, knowledge, agreement with delivering in a health facility and positive self-efficacy to deliver in a health facility were not statistically different at baseline and endline between study groups. However, there was a difference with perceived norms whereby more women at baseline in the comparison group were more likely to agree that women in the community delivered in a health facility (62.3% vs 72%). At endline, we found this association was no longer significant.

Behavioral determinants (e.g., knowledge that babies should be fed exclusively for six months, attitudes that babies were less likely to get sick if exclusively breastfed, etc) were not statistically different between study groups at baseline but were at endline. All behavioral determinants related to breastfeeding were positively associated with the intervention group at endline and all but self-efficacy remained statistically significant when controlling for study group and time.

For handwashing behaviors, attitudes and self-efficacy at baseline were statistically higher in the comparison group compared to the intervention group. However, this changed at endline where the intervention group was statistically higher for both attitudes and self-efficacy. For knowledge, knowing 3 critical times to wash hands was statistically significantly higher in the intervention area (44.1% vs 35.9%). This association continued at endline. When assessing the interaction term, we found that positive attitudes and perceived norms were positively statistically associated with study group and time while knowledge was not.

These findings were further supported in the qualitative data which highlighted how RFSA programming focused on strengthening knowledge across a range of health outcomes including behaviors such as exclusive breastfeeding which protects the health of the child while also preventing a woman from getting pregnant.

*"From Exclusive Breastfeeding, what we’ve learned from exclusive breastfeeding, as soon as the child is born, from birth until 6 months to give him only milk. Do not give him water or anything else, only milk.……. Its importance, it protects, it protects… .……*. *it protects the woman from getting pregnant… .If you respect the process… .It still protects your child from malnutrition*… . " *Woman Hamzari*

*FP*, *maternal health*, *nutrition*, *and WASH outcomes*

We estimated logistic regressions to determine the effect of the integrated SBC approach on the proposed outcomes. Table 3 presents the odds ratio of demographic and study characteristics for each study outcome. There are three main evaluation variables. “Group” examines differences between the intervention and comparison groups. “Time” examines differences between baseline and endline. The interaction of these two (group and time) measures whether healthy behaviors increased from baseline to endline more in the intervention than in the comparison group. There was not a statistically significant result in the interaction term, meaning that there was not a statistically observable effect of the intervention on the four health outcomes. However, across two of the four outcomes, we found that women who were exposed to messages were significantly more likely to have improved health outcomes. We found an increased odds of using modern contraception (Adjusted odds ratio (AOR) = 1.7, p < 0.001) and an increased odds of having a handwashing station (AOR = 1.4, p<0.01). For women participating in a RFSA sponsored women’s group, we found an increased odds of using modern contraception (AOR) = 1.4, p < 0.05) but a negative association with households having a handwashing station (AOR) = 0.6, p < 0.001).

**Table 3:** Adjusted odds ratio and confidence intervals of demographic characteristics and effect of intervention by health outcomes, Niger, 2021–2023.

Study characteristics	MCPR (N = 5,392)	Facility delivery (n = 5,225)	Exclusive breastfeeding (N = 1,411)	Handwashing station (N = 5,392)
*Group*: *intervention (reference*: *comparison)*	1.3 [1.0–1.7]*	1.2 [0.9–1.7]	1.0 [0.6–1.6]	1.0 [0.7–1.5]
*Time*: *endline (reference baseline)*	1.8 [1.4–2.4]***	1.6 [1.2–2.1]**	1.9 [1.2–3.2]**	0.5 [0.4–0.8]**
Interaction (time * group)	0.9 [0.6–1.2]	0.9 [0.5–1.4]	1.2 [0.6–2.1]	0.8 [0.5–1.3]
*Exposure to message (reference*: *no exposure)*	1.7 [1.4–2.0]***	1.0 [0.8–1.3]	1.1 [0.8–1.4]	1.4 [1.1–1.6]**
*Women’s group (reference*: *no participation)*	1.3 [1.0–1.6]*	1.1 [0.9–1.4]	1.1 [0.8–1.4]	0.6 [0.5–0.7]***
*Knowledge*	1.9 [1.4–2.5]***	1.6 [1.1–2.3]*	1.3 [0.9–2.0]	2.4 [2.0–2.8]***
*Attitudes*	1.2 [1.0–1.4]	1.1 [0.7–1.5]	0.8 [0.5–1.2]	0.4 [0.3–0.5]***
*Self-efficacy*	1.9 [1.6–2.5]***	9.2 [7.3–11.6]***	2.7 [2.0–3.6]***	0.7 [0.6–0.8]***
*Norms*	1.9 [1.6–2.2]***	3.1 [2.5–3.7]***	0.9 [0.7–1.2]	1.6 [1.2–2.0]***
*Wealth status (reference*: *poor)*				
Middle	1.3 [1.1–1.6]*	1.0 [0.8–1.2]	0.9 [0.7–1.2]	1.0 [0.8–1.2]
Wealthiest	2.1 [1.6–2.7]***	1.7 [1.4–2.1]***	0.8 [0.6–1.1]	0.7 [0.6–0.9]
*Experienced drought*	1.1 [0.9–1.3]	1.4 [1.1–1.8]**	0.8 [0.6–1.2]	0.5 [0.4–0.6]***
*Husband lives away*	0.7 [0.6–0.9]**	0.7 [0.6–0.9]*	0.7 [0.4–1.1]	0.6 [0.5–0.8]***
*Age group in years (reference 15–24)*				
25–34	1.1 [0.9–1.4]	0.8 [0.6–1.0]	0.8 [0.6–1.0]	0.8 [0.7–1.0]
35–49	1.0 [0.7–1.4]	0.9 [0.6–1.2]	0.6 [0.5–0.9]**	0.9 [0.7–1.2]
*Educational attainment* *(reference*: *no education)*				
Some formal schooling	1.3 [1.1–1.6]**	1.3 [1.0–1.6]*	0.9 [0.6–1.2]	1.0 [0.8–1.1]
*Marital status (reference*: *polygamous)*				
Married monogamous	0.9 [0.8–1.0]	1.0 [0.9–1.2]	0.9 [0.7–1.1]	1.1 [1.0–1.3]
*Listen to radio at least once a week (reference*: *less than once a week)*	0.9 [0.7–1.0]	1.0 [0.9–1.3]	0.8 [0.5–1.2]	0.8 [0.7–1.0]*
*Watch television at least once a week* *(reference*: *less than once a week)*	1.7 [1.3–2.4]***	1.6 [1.1–2.2]*	1.5 [0.9–2.5]	1.5 [1.0–2.3]

Significance

*p = < 0.05

**p = < 0.01

***p < 0.001.

For each health outcome, we used the corresponding knowledge, attitude, perceived norm, and self-efficacy measure described in Table 2. We observed statistically significant positive associations with knowledge and perceived norms for all health behaviors except for exclusive breastfeeding. We observed statistically significant positive associations with self-efficacy for all health behaviors except handwashing. We observed negative associations for attitudes and self-efficacy for the handwashing behavior.

### Contextual factors

We further explored contextual factors using qualitative data to understand how functionality of the health system and quality of care, evidence of climate related barriers, political insecurity, and the global Covid-19 pandemic may have influenced the program.

Respondents spoke about some of the challenges with the quality of health care provided. In some instances, respondents spoke about how health workers were not at the health facility or do not provide timely, quality care.

*"someone who brings a sick person, and the health workers stay in their sheds having their tea and look at you, the person accompanying the sick person has to talk to them, before anyone moves towards you*" *Woman Wadata*

While delays in program implementation occurred at the onset of the program due to the global Covid-19 pandemic, respondents at the community level did not mention Covid-19 as a challenge facing their communities. Rather infectious disease outbreaks due to measles and cholera continue to pose risks at the community level.

*"There haven’t been any other illnesses, but this year we’re a little bothered by Tininim [measles] disease among the children, even now*" *Male Wadata*

Respondents did not reflect on any challenges related to terrorism in the intervention zones despite prevalence in the Sahel region overall.

*"No, we don’t have any problems of conflict or violence in our village. Our main problem is famine*" *Female Wadata*

The qualitative evidence on climate related factors demonstrated links with drought and flooding and access to financial resources that could be used to access health services. Several respondents reflected on how a lack of rainfall or flooding contributed to poor crop yields and as a result there was insufficient food and crops to sell for money. The lack of income meant men would leave their families to search for income in Nigeria.

*"This drought has caused a lack of living…Which has meant that people don’t go to the health centers. You’re wasting time, because someone has to give it to you*… *Because it’s the fruits of the harvest that people sell… To have money to pay for consultations, it’s become so rare that no one would agree to sell" Female, Wadata*

*"Because if I have money on hand*, *I’ll call a motorcycle cab to take her straight to the health center*, *preferably treating an illness before it gets out of hand because taking too long to treat will cause double treatment*. *But if you don’t have the money*, *you have to go from house to house looking for a loan*. *But if [for example] the husband is absent*, *i*.*e*. *gone into exodus*, *waiting for him to send the money for the treatment*, *the situation will get even worse*." *Male*, *Girma*

Table 4 presents the REF results for the RISE II integrated SBC approach. Drawing from the program theory of change, we examine multiple sources of data to assess intervention exposure, behavioral and intermediate outcomes, and external factors and their contribution towards the program outcomes.

**Table 4:** Relevant explanation finder for the RISE II integrated SBC approach.

Description		Type	Level	Degree of influence	Implications
Alternative explanations & influencing factors	Mechanism				
If the RFSAs share health messages with women, then they will have the necessary information to improve health behaviors.	Sustained exposure to health messages will contribute to improved ideational determinants and health behaviors.	Primary explanation	Individual	Moderate. In two of the four outcomes (MCPR and presence of a handwashing station), we found positive associations between health message exposure and participation in a woman’s group. Qualitative evidence supports the explanation that the use of a cascade training approach helps to reach women and enables broader geographic coverage. However, volunteers also reflected on the challenges of sharing information with women without offering other forms of material support.	Message exposure in the three months preceding the survey and participation in a woman’s group enhances practice of some health behavior. RFSA activities that use a cascade training approach help to achieve a wider community of women through one-on-one communications.
If the RFSAs share health messages with families then they will have the necessary information to improve ideational determinants which will translate into improved support to women.	Sustained exposure to health messages for family members will contribute to improved support to women to practice health behaviors	Commingled rival	Interpersonal	Moderate. Qualitative evidence supported the explanation that RFSA activities encouraged family support for women particularly through activities that encouraged men to contribute to household responsibilities. However, women also reflected on the importance of income generating activities to achieve financial independence and decision-making autonomy.	RFSA activities that focus both on increasing male involvement in household responsibilities as well as increasing women’s financial autonomy through income generating activities are necessary.
If the RFSAs share health messages with community members, then they will have the necessary information to improve ideational determinants which will translate into improved support to women.	Sustained exposure to health messages for community members will contribute to improved support to women to practice health behaviors	Commingled rival	Community	Moderate. Qualitative evidence supports the explanation that community members were exposed to a health message through multiple channels beyond peer educators including radio, television, health workers. However, exposure may have been limited to poor access to radios in communities.	While mass media channels can reach a wide range of audiences across a broad geographic area, limited access to radio and television suggest programs tailored to the community context are needed to effectively address information needs.
If women have improved ideational determinants and family/community support, they will be more receptive/able to be practicing health behaviors	Improved ideational determinants and family/community support are associated with improved behaviors	Primary explanation	Individual	High. In three of the four outcomes (all but breastfeeding), we found knowledge, and perceived norms were positively associated with health outcomes. For MCPR and facility delivery, we found positive associations with self-efficacy. Qualitative evidence supported findings related to increased knowledge. A negative association was observed with self-efficacy to wash hands after defecating.	Higher levels of knowledge, attitudes, perceived norms, and self-efficacy observed in the intervention area at endline coupled with the statistically significant associations with health outcomes suggests progress towards improving health outcomes.
Availability of financial resources, quality healthcare and a secure environment are influencing factors	Availability of financial resources, quality healthcare and a secure environment can influence the implementation and outcome of the program	Implementation rival	Contextual	High. In three of the four outcomes (all but breastfeeding), we found when a partner does not live with the woman there is a negative association on the health outcomes. Increased wealth is associated with contraceptive use and facility delivery. Evidence from qualitative data suggests partner migration is a result of poor crop yields due to drought and flooding which requires men to move away in search of income. Drought is also negatively associated with presence of a handwashing station. We did not find significant evidence that Covid-19 and political insecurity had a negative impact on the program.	The implementation of the program is challenged by poverty and husbands moving away in search of income as this leads to reduced support to women to practice health behaviors.

### Contribution story

Drawing from the REF, the study team generated a contribution story for the Integrated SBC activities implemented under RISE II in Niger. The program worked primarily at the community level by engaging directly through community-based groups with women and members of their family to provide information and support that would contribute to improved knowledge, perceived norms and self-efficacy enabling them to adopt healthy behaviors. Strengthened male involvement in household responsibilities contributed to an improved home environment where women felt supported. Income generating activities contributed to financial autonomy for women to access services. The cascade implementation approach also enabled the program to increase its reach. Evidence further supports that higher levels of knowledge, attitudes, perceived norms, and self-efficacy observed in the intervention area at endline shown in Table 2 coupled with the statistically significant associations with the behavioral determinants and health outcomes in Table 3 suggest that progress has been made as described in the theory of change towards improving health outcomes. Given there was only two years between the baseline and endline, continuing activities with sustained implementation of the integrated SBC approach at scale may result in a statistically significant change in health outcomes particularly with continued emphasis on income generating activities which may serve as a protective factor given the continued increase in male partners moving away in search of financial resources.

## Discussion

Guided by the RISE II integrated SBC program theory of change, our findings describe the mechanisms through which the RISE II program was associated with behavioral determinants and health behaviors over time among women of reproductive age in Niger as well as contextual factors that influence programmatic impact. We found that women in the RISE II intervention zone were not statistically significantly more likely to have improved health outcomes compared to the comparison area at endline. However, we did find that exposure to health messages and participation in women’s groups were positively associated with modern contraceptive use and presence of handwashing stations suggesting the program likely had some effect. Given the high levels of engagement by non-governmental organizations in the region as well as exposure to health messages in both the intervention and comparison areas, it is likely interventions by other partner organizations may have made it difficult to tease out the effects of the RISE II SBC approach.

The program sought to address multiple health outcomes using community-based peer groups (i.e. Care groups) and home-visits which enabled trained facilitators to share health messages with women who were pregnant or had a child under the age of two. Previous studies have found that the use of Care Groups and similar interventions can contribute to improvements in maternal, newborn and child health outcomes [23,24]. Consistent with previous studies, we found the approach contributed to statistically significant improvements at endline for behavioral determinants (knowledge, attitudes, norms, self-efficacy) and positive associations of these over time across all indicators, except for the presence of a handwashing station [25–27]. The results suggest improvements in the behavioral determinants described in the theory of change occurred and that continued programmatic application may lead to sustained improvements in health outcomes in the future. A plausible explanation for the decline in the handwashing measure may be because the baseline study occurred one year after the onset of Covid-19 when many organizations were stressing the importance of hygiene related behaviors. It is conceivable that declines in this outcome were due to waning programmatic emphasis on hygiene behavior over time. In addition, Niger’s national policy that provided free delivery care for pregnant women was suspended in 2021 and likely made it challenging to see improvements for facility delivery given this had previously been a positive contributor to maternal health outcomes [28]. Given the declines in facility delivery over the course of the evaluation, policy makers should ensure that facility delivery remains free to guarantee access.

An important component of the RFSAs approach was to encourage intra-couple dialogue through peer groups as a lever of behavior change to promote joint decision making for health behaviors. Male engagement activities under RISE II contributed to increased dialogue among families about health behaviors and helped foster a supportive environment for practicing health behavior. Consistent with previous studies, these findings suggest that emphasis on male engagement programming has the potential to provide direct and indirect benefits to women’s health by improving partner communication [25,29–33]. Similarly, the incorporation of income generating activities for women in RISE II activities also served as an important element of support for women by providing opportunities for financial autonomy and decision-making agency.

At the community level, RFSAs also worked with community leaders and through radio programs to reinforce health messages. However, limited access to mass media channels compromised the project’s ability to reach a broader audience. The role that the health facility and providers played in sharing health messages, however, proved promising. Previous studies have found leveraging health system interactions [34,35] and integrating demand creation program with supply side interventions can lead to improvements in health outcomes [36,37].

The CA approach also provided an opportunity to explore how contextual factors may have contributed to programmatic impact. While Covid-19 contributed to delays in programmatic implementation, Covid-19 was not a primary factor related to adoption of healthy behaviors for communities. Rather, climate related factors including droughts and flooding placed significant stress on household agriculture production early in the study contributing to an increase in male partner out-migration to search for income at endline. The increase in male partner out migration over the course of the study as well as its negative association with health outcomes requires further investigation both in terms of understanding the mechanisms through which it influences health outcomes as well as testing approaches that may mitigate the impact of its effects [38,39]. It will also be important to understand how women’s agency and autonomy evolves as household composition changes through male out-migration. The results also suggest that programs should account for increases in partner migration and tailor interventions to address women’s needs for resources and financial autonomy as they manage a household without their partner.

### Limitations

There are several limitations to acknowledge. First, due to Covid-19 the baseline survey was delayed one year after implementation began. Therefore, early improvements as a result may not have been captured. Second, it was challenging to find a sample that was not exposed in some way to health messages given the numerous nongovernmental organizations operating in the region. Multiple efforts have been made to document SBC efforts throughout Niger but have lapsed or are no longer available due to lack of funding and/or do not provide detail at the subnational level [40,41]. Providing an extensive mapping of SBC efforts in the RISE II comparison areas was also outside the scope of this evaluation. Therefore, we were unable to provide a complete description of SBC efforts in the active comparison outside the quantitative measures of exposure. Third the evaluation did not address process evaluation questions and therefore we cannot address elements of implementation strength which may have had an impact on the results given the challenging context. Finally, while the study endline took place in February 2023, program activities have been extended through July 2025 and are not accounted for in this evaluation.

## Conclusion

Guided by the integrated SBC program theory of change, we evaluated the RISE II program’s effect on behavioral determinants and health behaviors among women of reproductive age in Niger. The program utilized Care groups and home-visits to disseminate health messages, which were effective in improving behavioral determinants such as knowledge, attitudes, norms, and self-efficacy. However, declines in the presence of handwashing stations were observed, likely due to prior programmatic emphasis during Covid-19. Policy changes over the evaluation period related to facility delivery fee for service also likely impacted use of maternal health services. Male engagement activities and income-generating opportunities for women were highlighted as crucial elements contributing to positive health behaviors and women’s empowerment. Contextual factors such as male outmigration was associated with health outcomes, underscoring the need for tailored interventions to support women managing households independently. While the RISE II program demonstrated positive impacts on behavioral determinants, adaptation and sustained implementation at scale are required to ensure program effectiveness considering the contextual challenges in Niger.

## Abbreviations

CA: Contribution analysis
EA: Enumeration area
FP: Family planning
IPC: Interpersonal communication
MNCH: Maternal, newborn and child health
REF: Relevant Explanation Finder
RFSA: Resilience Food Security Activities
RISE: Resilience in the Sahel Enhanced
SBC: Social and behavior change
